# Effective Use of Plant Proteins for the Development of “New” Foods

**DOI:** 10.3390/foods11091185

**Published:** 2022-04-19

**Authors:** Hiroyuki Yano, Wei Fu

**Affiliations:** Food Research Institute, National Agriculture and Food Research Organization, Tsukuba 305-8642, Japan; fui528@affrc.go.jp

**Keywords:** gluten-free bread, soy noodle, vegan cheese

## Abstract

Diversity in our diet mirrors modern society. Affluent lifestyles and extended longevity have caused the prevalence of diabetes and sarcopenia, which has led to the increased demand of low-carb, high-protein foods. Expansion of the global population and Westernization of Asian diets have surged the number of meat eaters, which has eventually disrupted the supply–demand balance of meat. In contrast, some people do not eat meat for religious reasons or due to veganism. With these multiple circumstances, our society has begun to resort to obtaining protein from plant sources rather than animal origins. This “protein shift” urges food researchers to develop high-quality foods based on plant proteins. Meanwhile, patients with food allergies, especially gluten-related ones, are reported to be increasing. Additionally, growing popularity of the gluten-free diet demands development of foods without using ingredients of wheat origin. Besides, consumers prefer “clean-label” products in which products are expected to contain fewer artificial compounds. These diversified demands on foods have spurred the development of “new” foods in view of food-processing technologies as well as selection of the primary ingredients. In this short review, examples of foodstuffs that have achieved tremendous recent progress are introduced: effective use of plant protein realized low-carb, high protein, gluten-free bread/pasta. Basic manufacturing principles of plant-based vegan cheese have also been established. We will also discuss on the strategy of effective development of new foods in view of the better communication with consumers as well as efficient use of plant proteins.

## 1. Introduction

What is expected on food research? It is gratifying that our life expectancy has increased [1,2]. On the other hand, the matter of “healthspan-lifespan gap” has been pointed out [3,4]. Abundance of food (easy intake of calories) and comfortable life (physical inactivity) have caused the prevalence of lifestyle-related diseases such as diabetes [5] and sarcopenia [6]. Thus, low-carb, high-protein foods have a high demand [7]. By contrast, global shortage of meat is considered a problem. Growing world population and concurrent Westernization of the Asian diet, especially the development of meat-eating habits, have caused the global shortage of meat supply [8]. Additionally, some people do not eat meat because of their religion or lifestyle [9]. Moreover, growing awareness of animal welfare encourages the production of plant-based alternatives to farmed meat. However, a significant number of consumers do not accept the “protein shift” from animal to plant easily because of attachment to meat and unwillingness to change habits [10]. Thus, food researchers/suppliers are enthusiastic to develop high-quality plant-protein foods welcomed by the majority of consumers.

In the development of new foods, food allergy is one of the most critical issues to tackle [11]. Development of gluten-free foods is especially demanded because wheat allergy and celiac disease occasionally trigger fatal symptoms such as anaphylaxis [12]. Furthermore, such foods should not be low-quality ones that wheat-allergy patients or celiac sufferers “reluctantly” eat but high-quality ones everyone enjoys and are available anywhere. As such, even in the case of disaster, a person on a restricted diet need not have difficulty in obtaining suitable foods.

Food researchers have developed new foods that meet the broad variety of consumers’ expectations. To survey consumers’ preferences effectively, they have enhanced communication skills with customers. In parallel, they have upgraded the processing technologies to test the production of such foods to reflect the survey results appropriately. This review introduces successful examples of such foods: gluten-free bread/pasta and vegan cheese. The trial-and-error as well as strategic approaches exercised thus far provide us with clues for developing future foods.

## 2. Development of Gluten-Free Bread

### 2.1. Cereal Flour-Based Breads

**Figure 1:**
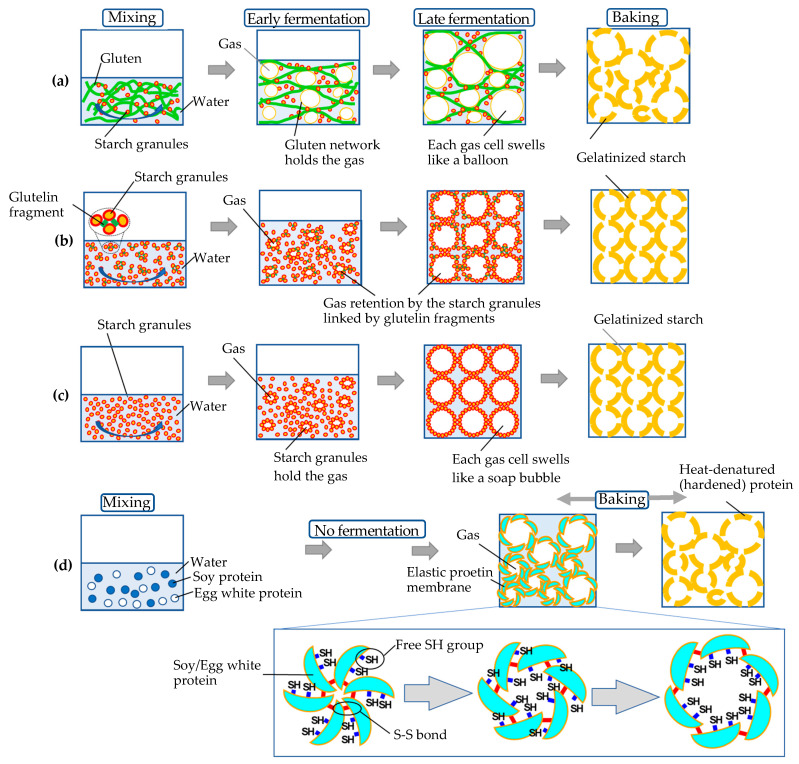
Comparison of the respective breadmaking processes: (**a**) conventional wheat bread; (**b**) protease-treated gluten-free rice bread [21]; (**c**) additive-free, gluten-free rice bread [22]; and (**d**) high-protein, low-carbohydrate bread made of soy protein and raw egg white [23]. (**a**,**c**) Adapted from references [24,25].

Archaeobotanical evidence shows that the origins of bread date back to 14,400 years ago in northeastern Jordan [13,14]. Since then, bread has always been one of the most popular foods of the world. Even today, the tantalizing aroma fuels consumers’ willingness to purchase bread [15]. While the COVID-19 pandemic affected the bread market, such as forcing bakeries to sell breads in an individually-wrapped manner [16], our love for bread has never diminished [17]. The manufacturing process of conventional wheat bread is summarized in Figure 1a. Mixing of wheat flour and water produces gluten, a viscous protein network capable of confining the fermentation gas produced by yeast. Thus, the fermenting dough swells like an assembly of small balloons. The membrane of each balloon is made mainly of gluten and starch granules. Baking the dough facilitates gelatinization of the starch and thus hardening of the membrane. Thus, the baked bread maintains air-celled structure in the crumb. However, celiac and wheat-allergy patients cannot eat bread [18]. Mostly, they are compelled to follow a gluten-free diet. Especially, the changes in daily life imposed by the pandemic reminded us of the difficulty in obtaining special foods, such as gluten-free or vegan dishes, in disaster [19,20].

Therefore, development of high-quality gluten-free foods or a vegan menu not particular to the celiac/wheat-allergy patients or vegans but for all consumers will raise the prevalence and availability. The main ingredient of gluten-free bread is non-glutinous cereals such as rice. Generally, a viscosity improver, exemplified by hydroxypropylmethyl cellulose (HPMC) or guar gam, is used as a substitute for gluten [26]. Rice batter with HPMC or guar gam swells in fermentation because the viscosity improver holds the gas similarly to gluten. Addition of transglutaminase to a gluten-free rice batter, along with HPMC [27] or guar gam [28], results in a higher specific volume of the resulting bread (HPMC, 2.7–2.8 cm^3^/g; guar gam, 2.0–2.1 cm^3^/g). Transglutaminase catalyzes the acyl-transfer reaction between the ε-amino group on protein-bound lysine residues and γ-carboxyamide group of protein-bound glutamine residues [29]. Thus, it is capable of introducing covalent crosslinking of proteins to make them form a gluten-like protein network [30].

On the other hand, since the first report by Renzetti and Arendt [31], protease has been utilized in gluten-free breadmaking [32]. Why, then, does the protease-added batter swell in fermentation and baking? Gas cell stabilization in protease-treated rice batter was further investigated by Hamada et al. [21]. Observation under the microscope revealed that in the protease-added batter, starch granules aggregated with partially degraded glutelin molecules working as “linker”. Moreover, retention of many small bubbles was observed during fermentation in the protease-treated stable batter as compared to large and irregular air bubbles in the collapsing control batter. The improved gas retention with yeast fermentation was related to a considerable reduction in sedimentation of the flour particles for the protease-treated batter [33]. Thus, the specific volume of the protease-added bread was 2.98 cm^3^/g, higher than that (1.36 cm^3^/g) of the control bread [21]. A hypothetical mechanism is illustrated in Figure 1b.

**Figure 2:**
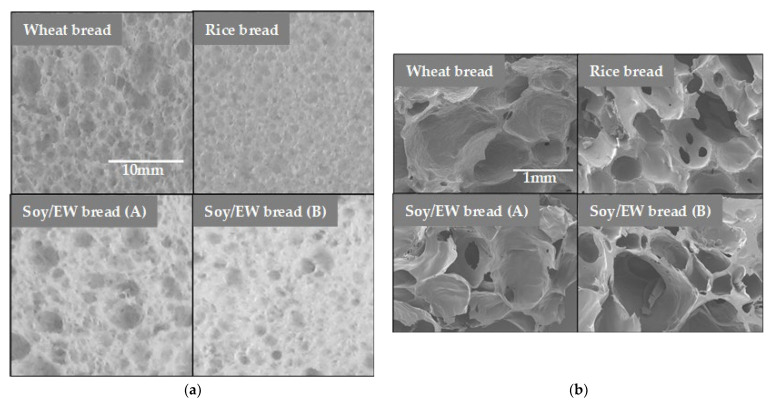
Cross-section of the breads. Observation under optical microscope (**a**) as well as under scanning electron microscope (**b**). Soy/EW bread indicates bread made of soy protein and raw egg white without (A) or with starch (B). Reprinted/adapted with permission from Ref. [23]. Copyright 2021, NARO.

However, some consumers claim that synthetic additives deteriorate the mouthfeel and/or the flavor of food [34,35,36,37]. Moreover, the clean-label trend [38] urges researchers to develop additive-free, gluten-free bread. Recently, such bread made solely of rice flour, water, yeast, sugar, salt, and oil has been reported [22]. The specific volume of the bread is as high as 4.0 cm^3^/g. The swelling mechanism was based on the Pickering foam (Figure 1c). Here, the starch granules of rice surround the fermentation gas. Thus, each gas cell swells like a soap bubble. Compared to the wheat dough, however, the additive-free rice batter in fermentation is fragile, like whipped meringue before baking. Therefore, some “tips for cooking” are required to make the bread successfully. First, rice flour with reduced starch damage should be used. Damaged starch tends to absorb water. When starch granules surrounding the fermentation gas absorb water, they cannot maintain the shape of each soap bubble; thus, the entire batter collapses. Second, the gas cells should be uniform in size. If the size of the cells is heterogenous, small bubbles tend to merge with the large ones, leading to increased instability of the batter. Therefore, the size of the air cells of the baked bread should be uniform (Figure 2a). It is quite a contrast to the crumb of the wheat bread composed of large and small air cells.

Further studies on the additive-free, gluten-free rice bread are in progress. Using nineteen rice flour samples containing amylose contents ranging from 9.6 to 22.3%, Aoki et al. [39] demonstrated that amylose content of rice flour positively correlated with the specific volume of the bread made from the flour. The specific volume of the bread reached to 5.0 cm^3^/g. They concluded that rice cultivars that have high amylose content and high ratio of opaque kernel grains can make high-quality rice flour and rice bread [40]. A similar tendency was observed for gluten-free bread in which carboxymethyl cellulose (CMC) was used as viscosity improver [41]. The additive-free, gluten-free rice bread and a breadmaker to make the bread have been on the market in Japan. The breadmaker KBD-X (Tiger Corporation, Kadoma, Osaka, Japan) has not only received good customers’ reviews at Amazon’s purchase site but also been used for gluten-free bread research [25,39,40,42].

Below, the ongoing and quite recent studies on the cereal flour-based gluten-free breads are introduced. *Usage of high-temperature water:* Non-additive GFB was also developed using thickening effect of starch gelatinization [43]. Addition of 70 °C water significantly improved the properties of bread (specific volume, 2.49 cm^3^/g; firmness, 1.55 N) compared to the addition of 5 °C water (specific volume, 2.05 cm^3^/g; firmness, 2.42 N). The authors claim that it is a cost-effective and simple method of producing bread from gluten-free rice flour by applying the thickening effect of gelatinization without the use of specific materials, such as thickening agents or rice flour with controlled starch damage. *Ohmic heating:* Optimization of the baking process may contribute to improved product quality. Ohmic heating of food products, achieved by passage of an alternating current through food, has emerged as a potential alternative to conventional heating. Key characteristics of ohmic heating are homogeneity of heating, shorter heating time, low energy consumption, and improved product quality and food safety [44]. A possible alternative to conventional oven baking is the use of ohmic heating in which fermenting batter is placed in an electric resistance oven [45]. Passage of electrical current through the batter generates heat as the batter functions as a resistance. Bender et al. [46] compared ohmic heating and conventional oven heating using the same batter composed mainly of buckwheat, wheat starch, egg albumen, and HPMC. Results showed that GF breads could benefit from the uniform rapid heating during processing, as these breads showed superior functional properties (specific volume, 2.86–3.44 cm^3^/g; relative elasticity, 45.05–56.83%; porosity, 35.17–40.92%) compared with conventional oven-baked GF bread (specific volume, 2.60 cm^3^/g; relative elasticity, 44.23%; porosity, 37.63%). Moreover, ohmic baking only requires a few minutes to obtain a fully expanded GF bread. Due to its volumetric and uniform heating principle, crumb development during baking and consequently bread volume is improved, which enhances the overall GF bread quality [47]. *Partial Vacuum Baking:* Baking GF bread under partial vacuum is worth trying. Because water’s boiling point decreases under reduced pressure, it is expected that its distribution within the batter and its interactions with the other constituents, mainly starch, would differ from those in bread baked under atmospheric pressure [48]. Şimşek [49] evaluated the impact of partial-vacuum baking (60 kPa vacuum pressure) on the quality and storage properties of gluten-free bread (GFB). No significant differences were observed in the specific volume and hardness of the partial-vacuum-baked bread (specific volume: 1.53 cm^3^/g; hardness: 17.82 N) compared to the control bread baked at atmospheric pressure (specific volume: 1.44 cm^3^/g; hardness: 20.12 N). However, the DSC, SEM, and XRD results showed that a more crystalline structure and different starch crystal types formed after partial-vacuum baking. Moreover, partial-vacuum baking significantly affected the total water loss and the texture parameters during storage for three days. Partial-vacuum-baked samples were softer and had a tendency to become stale more slowly than the control. The findings indicate that the partial-vacuum-baking method increases the shelf life of gluten-free products by modifying the microstructure of the bread.

### 2.2. Non-Cereal-Based High-Protein, Low-Carb Breads

Thus far, several efforts have been made to develop high-protein, low-carbohydrate bread whether wheat-based or gluten-free. These breads are mostly made by adding plant/animal protein, such as chickpea flour [50], pea protein [51], green lentil flour [52], microalgae [53], or cricket powder [54], to a wheat dough or rice-based batter (Table 1). However, it is not easy to add high proportion of protein while concurrently maintaining the volume of the cereal-based dough/batter in the fermentation process. In other words, adopting cereal flour as a major ingredient of the bread was the bottleneck in the development of protein-rich bread.

**Figure 3:**
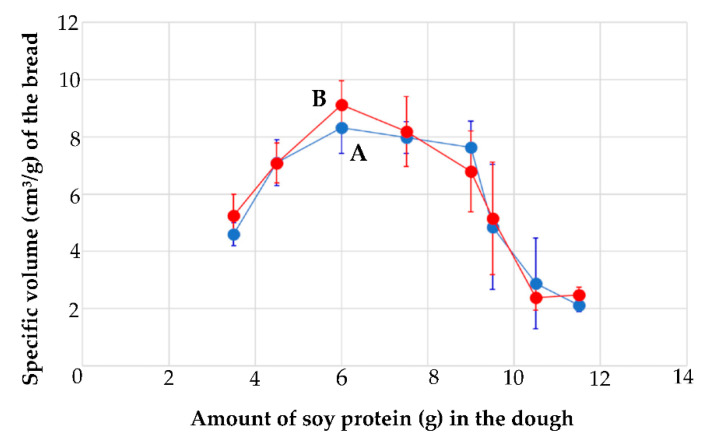
Specific volume (cm^3^/g) of the bread made of soy protein and egg white protein. Horizontal axis shows the amount (g) of soy protein added to 22.5 g of a raw egg white in making the dough. (A) No starch and (B) cornstarch (2.5 g) were added to the dough, respectively. Reprinted/adapted with permission from Ref. [23]. Copyright 2021, NARO.

Recently, development of a high-protein/low-carbohydrate bread was reported [23]. A dough made of soy protein isolate (SPI) and raw egg white (EW) is baked in an oven without the need of fermentation process (Figure 1d). Figure 3 shows the specific volume of the bread. Blue circles show the bread volume of the starch-free bread, while the red ones show starch-added bread. The horizontal axis is the amount of the SPI (g) against the raw EW (22.5 g) in the dough. The vertical axis indicates the specific volume (cm^3^/g) of the bread made from the dough. The maximum specific volume is over 6 cm^3^/g, comparable to that of the wheat bread. However, the error bars show that controlling the specific volume, that is, stabilizing the swelling ratio of the bread, remains an issue.

**Figure 4:**
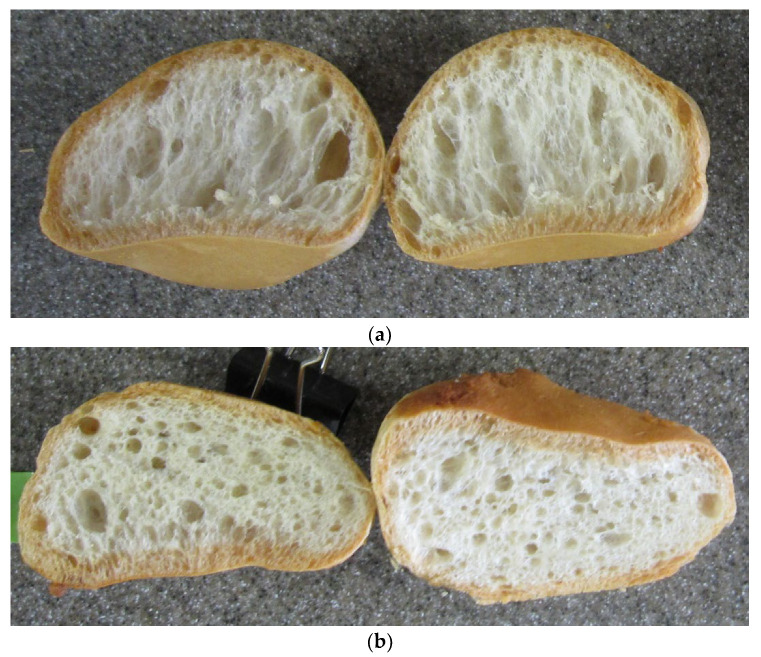
Half-cut end surface of the bread made of soy protein/raw egg white. (**a**) No starch and (**b**) cornstarch (2.5 g) were added to the dough, respectively. Reprinted/adapted with permission from Ref. [23]. Copyright 2021, NARO.

Table 2 compares the nutrition information with other breads. The protein content is higher, and the carbohydrate content is lower than the cereal-based breads. The cross-section of the breads (Figure 4) shows that it has air-celled structure like other cereal-based breads. The sizes of the gas cells are not uniform. Therefore, the cell structure is more like that of a wheat bread than that of the rice bread (Figure 2a and Figure 4).

Figure 2b compares the microstructure of the breads’ crumb under the scanning electron microscope (SEM). It is noteworthy that while the breads were made based on the respective mechanisms, they shared similar basic microstructure. However, the surface of the crumb of the wheat bread made from gluten and starch granules looked “rough”, supposedly due to the trace of starch granules. Additive-free, gluten-free rice bread mostly made of rice starch granules showed a similar appearance. In contrast, the surface of the breads’ crumb made from soy protein and raw egg white looked “smooth”, probably because they did not contain any structured materials such as starch granules. Sensory evaluation and quality improvement of the breads are in progress in our lab.

Next, we ask why the soy protein/egg white dough swells upon heating. The mechanism of “aburaage” making is the clue. Aburaage, a fried tofu, is a traditional Japanese cuisine that attracts attention as a healthy soy-based product [55]. Upon frying in oil, tofu expands, resulting in a spongy structure with a crispy, golden-colored skin. The mechanism of the expansion has been explained by a successive S-S (disulfide)/SH (sulfhydryl) exchanges among the soy proteins in the heating process [56]. It is well-known that gelation property of soy protein depends on temperature [57]. Around 100 °C to 140 °C, hard/fragile soy protein becomes soft/elastic with the ability to form air cells. Hashizume explained that the dissolved air bubbles in tofu work like a boiling stone in boiling water during the frying, and the growing bubbles serve to expand the interspaces of protein gel, resulting in the well-expanded aburaage [56]. Recently, it was reported that addition of the EW protein to SPI significantly increased the number of intermolecular disulfide linkage in which egg white protein serves as a free sulfhydryl (SH) donor [58]. Thus, in the case of the SPI/EW bread, the soft/elastic membrane composed of soy/EW protein managed to hold the gradually expanding air by continuously exchanging the S-S/SH structure. A hypothetical model is provided in Figure 1d. Elucidation of the mechanism is in progress in our lab.

## 3. Development of Gluten-Free Pasta

Progress in the development of gluten-free pasta is briefly mentioned here. Recent reviews by other authors cover history, classification, materials, and processing of Asian noodles [59] as well as efforts to improve nutritional value of pasta [60]. 

**Figure 5:**
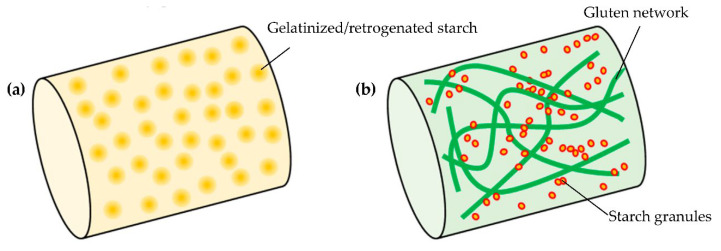
Schematic diagram of gluten-free pasta (**a**) and wheat pasta (**b**).

Generally, dough for noodles is made using water and cereal flours, such as wheat and rice, which are mixed and shaped into long and thin structure to make fresh pasta (Figure 5). In the case of wheat pasta, the protein network of gluten and wheat starch granules sustain its structure and physical property. The protein–starch matrix also plays a key role in its digestibility [61]. For gluten-free rice pasta, a viscosity improver, such as HPMC or guar gam, is occasionally used as a substitute for gluten [62]. In the case of the additive-free, gluten-free rice pasta [63], gelatinized/retrograded starch works as its framework. Comparative schematics of wheat pasta and sorghum gluten-free pasta noodle structure are provided in a recent review [64], which is useful to understand the respective three structures. As with the case of bread, fortification of pasta with exogenous protein, such as cricket [65], Brewer’s spent grain [66], and rice protein [67], was reported. These trials are useful to increase nutritional values other than protein, such as fiber and minerals. Some are economical utilization of by-products, which also contributes to SDGs.

Recently, a protein-rich pasta made exclusively of yellow pea and water was reported [68]. In terms of the physical property, the extrusion-cooked pasta had high values for both breaking stress and breaking strain and was highly evaluated in the hedonic sensory tests. Moreover, it has a low glycemic index (50.4) as well as insulin-saving properties. Thus, it has potential as a functional staple food. The pasta is commercialized under the name of “Zenb pasta” from Mizkan Holdings Co., Ltd. (Handa, Aichi, Japan), and is available on the Internet. Then, what is the mechanism to make pasta exclusively from pea without additives? Pea protein changes its structure in heating/mixing [69]. During heating, the dissociation of legumin and their rearrangements via hydrophobic interactions and sulfhydryl (SH)/disulfide bonds (S-S) reactions occur, and they are considered to result in the formation of high-molecular weight aggregates of random structure. Subsequent cooling process renders cold gelation of pea protein in which these aggregates assemble into a structured network by lowering electrostatic repulsions. Thus, extrusion processing allowed pea protein to construct a resilient network similarly to wheat gluten, realizing the pea-based pasta. 

Extrusion cooking expanded the consumption of pulses [70]. Moreover, Tao et al. [71] reported morphing pasta structure by a low-cost manufacturing method based on a simple and universal diffusion-based mechanism. These innovative processing techniques will produce new pasta with high quality, meeting the huge variety of consumers’ demands.

## 4. Plant-Based Cheese

### 4.1. Conventional Milk Cheese

**Figure 6:**
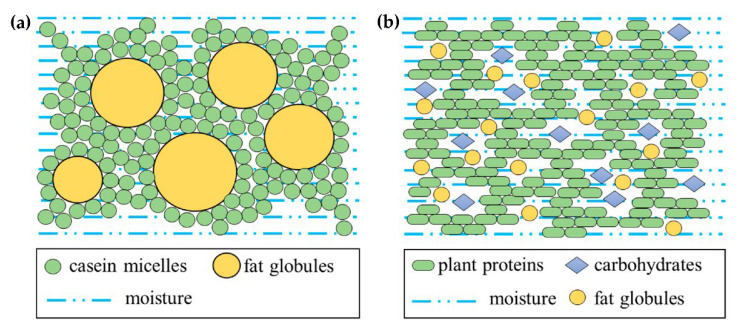
Comparison of the structure of the respective cheeses: (**a**) conventional milk cheese and (**b**) plant cheese.

Conventional milk cheese, generally perceived as natural cheese, was born from an accidental curdling of milk. Casein is the main protein in milk. It is hydrophobic and exists as casein micelles. Cheese curdling is decided mainly by this hydrophobic force of casein micelles forming a network structure [25]. In conventional milk cheese, casein micelles and fat globules are two main components contributing to create the structure that stretches throughout cheese (Figure 6a). Commonly, casein micelles are considered as the continuous phase and fat globules as the disperse phase, whose concentration and state directly relate to cheese structure and could contribute a wealth of texture properties. 

Because of the constant pursuit and innovation of cheese making, the original milk cheese was derived from the traditional sense of fermented natural cheese to multiple types of cheese products, such as processed cheese, cream cheese, and cheese analogues, which has led to a diverse and complex structure in cheese. The most common and well-developed cheese product is processed cheese. Processed cheese mainly consists of natural cheese, emulsifying salts, water, and limited amounts of additional ingredients, which are developed based on emulsification. It was clarified that emulsifying salts, water, and additives, such as pre-cooked cheese or starches, had obvious impact on the textural properties of processed cheese. Factually, the changes in textural properties of cheese are substantially affected by casein network structure and fat globules. It was demonstrated that a sufficiently emulsifying with high stirring speed and long stirring time could reduce the sizes of fat globules and cause the casein network structure to become fine-stranded [72,73,74,75].

### 4.2. Plant Cheese

Recently, plant cheese is coming to the forefront of consumers’ minds [76]. There has been a growing trend towards the cheese analogues manufactured from plant-based ingredients to produce a conventional cheese-like product to meet specific requirements. Plant cheese is required to develop as close as possible to conventional cheese in terms of texture, taste, nutritional value, and appearance. The key point is to construct a structure sufficient to express the smooth and uniform texture by using effective plant components. In actual development of cheese analogues containing multi-components and multi-phases, major hydrocolloids (proteins, carbohydrates), water, fat, and other components (e.g., salts, minerals, and functional substances, etc.) play critical roles in conferring texture and stability. Moreover, interactions of hydrocolloids with other food components also govern the network strength of cheese. Therefore, texture design of plant cheese needs to consider functional attributes of selected ingredients and assemble the different ingredients together to create a balanced structure for desirable texture. In plant cheese, plant proteins, carbohydrates, and fats may be linked together to increase the interactions by emulsions (Figure 6b). 

There are primarily two types of approaches in plant-cheese manufacturing [76]. Firstly, plant cheese can be made by blending individual components, including plant proteins and fats. As a core, proteins build structures and provide mechanical strength, and biochemical processes (enzymes) can also be carried out through proteins as the medium. Another critical role of the proteins is their ability to adsorb to surfaces and form protective coatings. These proteins have an amphiphilic character, which allows the hydrophilic part to adsorb to water and the hydrophobic part to adsorb to oil or air [77]. The prime ingredient of plant protein could be protein powder, and even plant-based milk could also be used. In recent years, the consumption of soy milk has increased for plant-cheese making because of its remarkable nutrient profile, which is the closest to milk [78]. Secondly, starches have also been most commonly used to control mechanical properties and stability of plant cheese, for which gelatinization upon heating and cooling is important to the thickening and gelling properties [79]. Commonly, tapioca, potato, and corn starches are used in plant-cheese formulation. The behavior of starches in plant cheese is governed by the precise molecular characteristics of the amylose and amylopectin as well as their ratio [80]. Fats are also used to determine the desirable appearance and texture of plant cheese equally as proteins and carbohydrates. Fat globules occupy space in the protein matrix and prevent a dense network that results in a hard and corky texture. Fats also disturb the flow of fluids inside the plant cheese when they are stirred, making plant cheese feel thick [77]. Processing technologies such as shearing, emulsification, and thermo-extrusion can also improve the formation of texture in plant cheese. These processes enable the transformation of protein-native structures into an unfolded and denatured form to promote the interaction between proteins and carbohydrate polymers [81]. 

## 5. Communication with the Consumers

In developing new food products, communication with consumers is critical to meet their demands. To obtain consumer-based perceptual product profiles, the Check-All-That-Apply (CATA) questionnaire has become a popular method for its convenience and effectiveness [82]. Explained briefly, the CATA method consists of two procedures. First, from the list of easily understandable texture terms, each consumer panelist selects appropriate terms to describe each food sample without any limitation on the number of selected terms. This test is conducted for all samples, for example, three breads. Subsequent cross-tabulation and correspondence analyses portray each sample’s perceptual product profile from consumers. A comparative profile of the samples is provided on a single two-dimensional map, proposing two critical axes (for example, soft/moist vs. hard/dry as well as fine vs. coarse of the bread crumb) to evaluate the product samples [25]. The simplicity and effective performance of CATA have made it popular and widely used in the research and development of gluten-free breads [83] as well as vegan spread products [84]. Not only is it useful for a simple comparison of bread samples, such as appearance, flavor, and texture [85], but it is also applicable to a seemingly complicated study, such as comparison of oral processing behavior between gluten-free or gluten-containing breads [86]. The gluten-free bread was perceived crumbly, dry, and sandy and had a longer eating duration than the gluten-containing bread, which was perceived soft, spongy, pasty, and sticky. The structure of the GF bread was easily fragmented during mastication, and a longer period in the mouth was required to prepare a cohesive bolus for swallowing. The addition of spreads, such as butter or mayonnaise, did not alter moisture and oral processing behavior, but it increased the softness perception. Therefore, the investigation of oral processing behavior using CATA analyses has provided valuable information on the relationship between food structure and perception, a useful clue for the design of gluten-free bread formulations. 

Moreover, friendly and accessible tools with a sense of fun have come into popular use. “Emoticons” are pictorial/textual depictions of facial expressions recently attracting attentions in marketing communications [87]. A combination of CATA/emoticon along with the instrumental physical measurements was successfully applied to identify quality parameters of gluten-free breads and how these influence liking, softness, and emotions terms described by consumers [88]. This may work as a promising predictor correlating dough parameters, bread physical properties, and sensory quality of the breads. It helps food scientists and producers to establish whether the trial products meet consumer expectations.

Furthermore, effective use of familiar tools such as Twitter helps researchers understand consumers’ attitudes. Over 120,000 tweets relating to veganism were extracted from Twitter, which were then analyzed using a text analytics tool to ascertain the predominant themes of conversation taking place around vegan food [89]. Value propositions communicated with respect to personal health attributes (e.g., dairy free, gluten free, and nutrition) and consumption benefits (e.g., tasty, delicious) have been shown likely to resonate with consumers and motivate increased consumption while concurrently delivering environmental benefits as a positive side effect. Effective use of these familiar tools makes the relationship between researchers/producers and consumers closer, helping to realize better communication, strategic product development, as well as success in business.

## 6. Shift from Animal to Plant Proteins

Effective use of plant protein in the development of high-protein, low-carbohydrate foods not only complements over-demanded animal protein but also contributes to sustainability [90]. Indeed, a variety of plant proteins, such as sunflower, walnut, hemp, grapeseed, and sesame, are economical uses of waste and byproducts generated in the production of vegetable oils [91]. Thus, their usage will continue to expand in relation to SDGs [92]. Mutual interaction of protein molecules [93] as well as interaction between protein and amylose/amylopectin molecules [94] in food processing, especially in the extrusion, plays important roles in the physical property of foods. In particular, linkage/cleavage of the disulfide (S-S) bonds causes drastic change of the protein structure [95] as observed in both plant physiology [96,97] and in food processing [98,99]. Extrusion heating, pressing, as well as mixing of a variety of plant proteins facilitates the regulation of the disulfide structure, thus playing critical roles in building the strategy to obtaining the desired property [100]. Soy protein has been extensively used as a major ingredient in plant food, with its excellent technological property and health benefits, such as reducing the risk of cardiovascular disease, hyperlipidemia, heart disease, diabetes, osteoporosis, and breast cancer mortality [101]. Recently, maintenance of satiety after ingestion of soy has also been reported [102]. Thus, soy meat and other soy-based products have led the plant food market. In Europe, the plant-protein market is projected to reach USD 3.8 billion by 2026, at a CAGR of 6.66% during the forecast period, 2021–2026 [103].

Other plant proteins are attracting attention as well. Due to its hard-to-cook characteristics, mung bean had remained underutilized. Recently, however, by optimizing the extrusion parameters, texturized mung bean protein with desirable physical properties has been successfully produced as a candidate for meat extender [104]. Moreover, microstructure analysis revealed a fibrous structure that is aligned along the shear flow, while partial protein unfoldment was observed that is crucial for protein fibril formation during texturization. The pilot-scale basis study, from initial protein extraction up to final extrusion step allows food industry to readily adapt the processing parameters for customized production of texturized mung bean protein. Utilization of sesame [105], faba bean [106], pea [107], rapeseed [108], sugar beet [109], lentil [110], cotton seed [111], and other plant proteins are in progress. Furthermore, spirulina, a photosynthetic Cyanobacteria, has been available as a nutraceutical food supplement, as its extract contains functional ingredients such as phycocyanin, phenols, and polysaccharides, with anti-inflammatory, antioxidant, and immunomodulating properties [112]. Because of the high protein content (60–70%) of the dry weight as well as its high digestibility [113], spirulina is utilized for meat analogue [114] and snacks [115]. Recently, it has attracted attentions as a natural drug, as it exerts anti-inflammatory and antioxidant effects, acting on glial cell activation and in the prevention and/or progression of neurodegenerative diseases, in particular Parkinson’s disease, Alzheimer’s disease, and multiple sclerosis [112].

On the other hand, some reports raise alarm on a rapid dietary shift from animal to plant protein [90,116]. Thus, a balanced diet composed of both animal and plant proteins is ideal. In the environment of the shortage of chicken, porcine, and cow meat, insect protein is one of the options. Because of its high nutritional quality [117] as well as gradual acceptance by consumers [118], insect protein will expand its territory. Other animal protein sources, such as crayfish [119] and jellyfish [120], look promising. Cell-based meat is another option. Animal cells rather than slain livestock are utilized for cell-based meat production, where the obtained products resemble traditional meat from an animal. The research/business of the cell meat has already started and will likely continue to build momentum in the next decade [121]. 

To tackle the global-scale challenges such as protein shift, a food-processing sector supported by the government is indispensable. For example, in the Netherlands, the region of Wageningen has developed a food innovation cluster that involves a large number of food firms, global agri-food incumbents, and leading knowledge and educational institutions, including Wageningen University and Research Center [122]. The Foodvalley leads studies on food sciences, including the development of plant-based foods. There, research institutions and food companies work together seamlessly from basic research to industrialization. 

Rapid progress in food-processing technology has realized new plant-based foods almost identical to the original, animal-based ones: plant meat with realistic fiber [123], plant patties whose color changes red to brown by heat [124], and vegan eggs with runny egg white and yolk [125]. Before long, at a restaurant, we may trim off elaborately made fat and gristle from a plant steak full of fake blood. On another day, plant-made fishbone may get stuck in a consumer’s throat. In due course, however, children who have never seen animal meat might appear, just as children today are unfamiliar with CRT-based television, cassette tapes, or floppy disks. When such a time comes, food researchers might stop mimicking the original “antique” foods and start developing simply nutritional, ecofriendly new foods. 

## 7. Conclusions

Rapid growth of human population, global warming, and animal welfare have inspired a protein shift from animal to plant. Rich and aging societies now encounter lifestyle-related diseases, such as diabetes and sarcopenia, which urge food researchers to develop high-protein, low-carbohydrate foods. Celiac/wheat-allergy patients are compelled to follow a gluten-free diet. Thus, food researchers and suppliers have been making efforts to meet multiple consumer needs. Evolution of the food-processing technology has realized authentic-looking gluten-free bread/pasta, plant cheese, soy meat, and other plant-based products. In parallel, researchers have enhanced communication skills with consumers by taking advantage of friendly tools, such as emoticons and Twitter. Effective utilization of a variety of plant proteins will realize development of new foods highly-accepted by consumers with their high nutritional value. Then, what comes next? In the near future, researchers are developing innovative foods by not mimicking but surpassing the original food products unintentionally. 

## Figures and Tables

**Table 1:** Recent examples of cereal-based high-protein, low-carb breads.

Protein Source	Main Cereal Flour	Protein Content (g/100 g)	Specific Volume of Bread (cm^3^/g)	References
Chickpea	Rice	12.0–13.2	2.5–2.9	[50]
Pea	Buckwheat/ Flaxseed	17.1	1.8–2.5	[51]
Lentil	Wheat	10.3–12.4	3.4–3.5	[52]
Microalgae	Buckwheat/Rice flour, Potato starch	7.2–7.9	2.0–3.0	[53]
Cricket	Corn/Potato	8.5	-	[54]

**Table 2:** Comparison of the nutrition composition among the breads.

	Wheat Bread	Rice Bread	Soy/EW (A)	Soy/EW (B)
Water (g/100 g)	36.8	40.5	44.9	41.6
Protein (g/100 g)	9.5	3.8	49.6	42.8
Lipid (g/100 g)	5.3	2.4	2.4	2.5
Ash (g/100 g)	1.4	1.2	2.4	2.1
Carbohydrate (g/100 g)	47.0	52.1	0.7	11.0
Energy (kcal/100 g)	269	243	224	234
Salt (g/100 g)	1.2	1.0	1.72	1.5

Reprinted/adapted with permission from Ref. [23]. Copyright 2021, NARO.

## Data Availability

Not applicable.
